# Complete Genome Sequence of Stenotrophomonas maltophilia Siphophage Sonora

**DOI:** 10.1128/mra.00167-22

**Published:** 2022-03-23

**Authors:** Michael Teve, James Clark, Tram Le, Ben Burrowes, Mei Liu

**Affiliations:** a Department of Biochemistry and Biophysics, Texas A&M University, College Station, Texas, USA; b Center for Phage Technology, Texas A&M University, College Station, Texas, USA; c BB Phage Consultancy, LLC, Georgetown, Texas, USA; Queens College CUNY

## Abstract

Phage Sonora is a siphophage that was isolated against the opportunistic human pathogen Stenotrophomonas maltophilia. The genome of phage Sonora is 63,825 bp long and is not related to that of any phage at the nucleotide level. Sonora shares 46 of 97 total proteins with the *Bordetella* phages CN2, MW2, and FP1.

## ANNOUNCEMENT

Stenotrophomonas maltophilia is an opportunistic human pathogen that is naturally found in water and soil environments (1). Some infections caused by S. maltophilia are difficult to treat due to the multidrug resistance of the causative strains. Because antibiotic resistance remains a significant public health issue, alternative treatments, such as phage therapy, to combat these infections are of interest. Bacteriophage Sonora’s isolation and genome characteristics are described in this report.

Sonora was isolated in 2019 from a topsoil sample (collected ∼3 in. from the surface) collected in Caldwell, Texas (coordinates: 30.612660, −96.551836). The phage was isolated and purified following the soft agar overlay method (2) using S. maltophilia (ATCC 51331) as the host, cultured aerobically at 30°C in tryptone nutrient (0.5% tryptone, 0.25% yeast extract, 0.1% glucose, 0.85% NaCl [wt/vol]) broth or agar. Sonora’s genomic DNA was extracted using a modified Wizard DNA cleanup kit protocol as described (3). DNA libraries were prepared as 300-bp inserts using a Swift 2S Turbo kit and sequenced on an Illumina MiSeq sequencer with paired-end 150-bp reads using 300-cycle v2 chemistry to produce 242,366 raw reads. These reads were quality controlled using FastQC (www.bioinformatics.babraham.ac.uk/projects/fastqc), trimmed with FASTX-Toolkit v0.11.6 (http://hannonlab.cshl.edu/fastx_toolkit/download.html), and assembled with SPAdes v3.5.0 (4) into a raw contig with 140-fold coverage. Since the raw contig assembled by SPAdes is usually opened at a random spot in the middle of the genome and the contig ends often have redundant or missing bases, PCR amplification of the genomic DNA using primers designed off the contig ends, followed by Sanger sequencing of the PCR product, allows verification of sequences in that region. Sonora’s genome was completed by PCR and Sanger sequencing using the primers 5′-ACTACCACGGTCACGCATAC-3′ and 5′-CAGATCATCGAACATGCCGC-3′. Genome termini were predicted by PhageTerm (5). The genome was annotated with the Center for Phage Technology (CPT) Galaxy-Apollo platform (https://cpt.tamu.edu/galaxy-pub) (6–9); structural annotation was performed using GLIMMER v3 (10) and MetaGeneAnnotator v1.0 (11) to predict protein-coding genes, with manual adjustments as needed. Rho-independent termination sites were identified using TransTerm (http://transterm.cbcb.umd.edu). The functional annotation of Sonora utilized InterProScan v5.48 (12), BLAST v2.9.0 (13), TMHMM v2.0 (14), HHpred (15), LipoP v1.0 (16), SignalP v5.0 (17), and SwissProt (18) databases. Sonora’s genome DNA sequence similarity to other phages was calculated by progressiveMauve v2.4 (19). All software was used with default settings.

**FIG 1:**
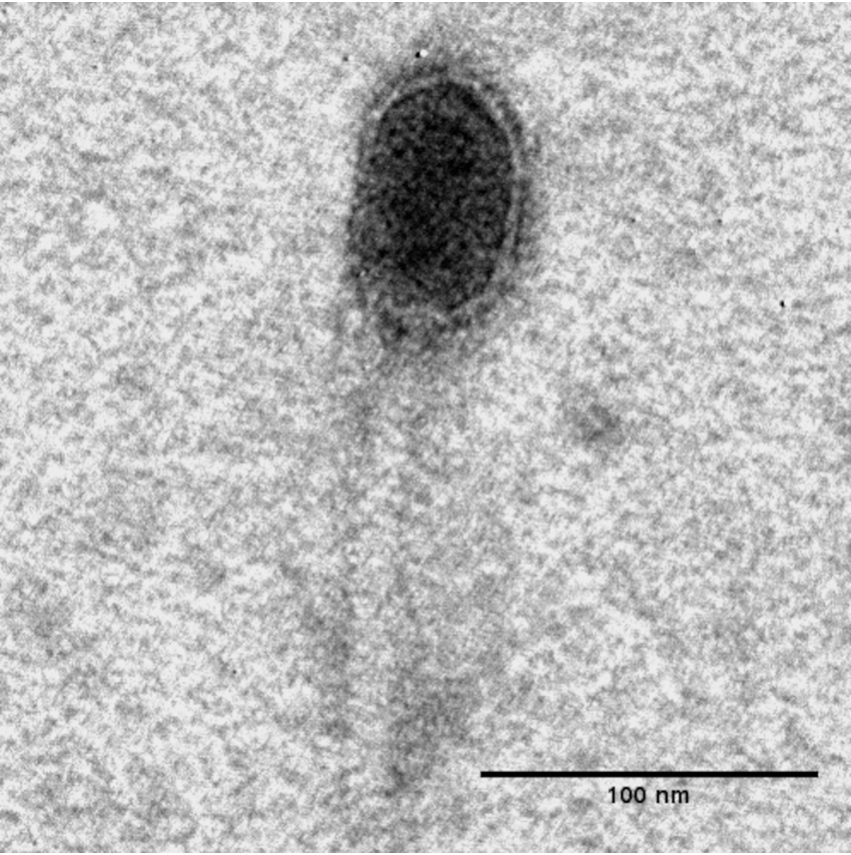
TEM of phage Sonora. Phage particles were diluted with TEM buffer (20 mM NaCl, 10 mM Tris-HCl [pH 7.5], 2 mM MgSO_4_) and captured on a freshly glow-discharged, Formvar carbon-coated grid. The grids were stained with 2% (wt/vol) uranyl acetate and observed at 100-kV accelerating voltage on a JEOL 1200 EX system at the Microscopy and Imaging Center at Texas A&M University.

Phage Sonora was determined to be a siphophage via negative staining with 2% (wt/vol) uranyl acetate and imaging by transmission electron microscopy (TEM) at the Texas A&M Microscopy and Imaging Center (Fig. 1). Phage Sonora has a genome of 63,825 bp, with a coding density of 93.8% and a G+C content of 63.0%. PhageTerm predicted a *cos* sequence at position 46430, which is within a gene encoding a predicted membrane protein. However, this location is separated by ∼18 kb from the gene encoding the terminase small subunit, suggesting that the packaging signal is significantly separated from the gene encoding the protein that recognizes it for packaging. A lysis cassette containing a transglycosylase endolysin with a signal arrest release (SAR) domain, a class I holin, and an inner and outer membrane spanin complex, with the o-spanin gene completely embedded within the i-spanin gene, was identified. Sonora is not closely related to any phages in the NCBI database, sharing at most only 33 to 34% genome-wide nucleotide sequence identity with several phages, such as Pseudomonas phage YuA (GenBank accession number AM749441) and *Bordetella* phage CN1 (GenBank accession number NC_047876), as determined by progressiveMauve (19). At the protein level (BLASTp, with E values of <0.001), Sonora shares 46 of 97 total proteins with each of the three *Bordetella* phages, namely, CN2 (GenBank accession number NC_047877.1), MW2 (GenBank accession number NC_047879.1), and FP1 (GenBank accession number NC_047878.1).

### Data availability.

Sonora’s genome was deposited in GenBank with accession number MZ326860. The associated BioProject, SRA, and BioSample accession numbers are PRJNA222858, SRR14095252, and SAMN18509468, respectively.
